# CoDNet: controlled diffusion network for structure-based drug design

**DOI:** 10.1093/bioadv/vbaf031

**Published:** 2025-02-19

**Authors:** Fahmi Kazi Md, Shahil Yasar Haque, Eashrat Jahan, Latin Chakma, Tamanna Shermin, Asif Uddin Ahmed, Salekul Islam, Swakkhar Shatabda, Riasat Azim

**Affiliations:** Department of Computer Science and Engineering, United International University, Dhaka 1212, Bangladesh; Department of Computer Science and Engineering, United International University, Dhaka 1212, Bangladesh; Department of Computer Science and Engineering, United International University, Dhaka 1212, Bangladesh; Department of Computer Science and Engineering, United International University, Dhaka 1212, Bangladesh; Department of Computer Science and Engineering, United International University, Dhaka 1212, Bangladesh; Department of Computer Science and Engineering, United International University, Dhaka 1212, Bangladesh; Department of Electrical Computer and Engineering, North South University, Dhaka 1229, Bangladesh; Department of Computer Science and Engineering, Brac University, Dhaka 1212, Bangladesh; Department of Computer Science and Engineering, United International University, Dhaka 1212, Bangladesh

## Abstract

**Motivation:**

Structure-based drug design (SBDD) holds promising potential to design ligands with high-binding affinity and rationalize their interaction with targets. By utilizing geometric knowledge of the three-dimensional (3D) structures of target binding sites, SBDD enhances the efficacy and selectivity of therapeutic agents by optimizing binding interactions at the molecular level. Here, we present CoDNet, a novel approach that combines the conditioning capabilities of ControlNet with the potency of the diffusion model to create generative frameworks for molecular compound design. This proposed method pioneers the application of ControlNet in diffusion model-based drug development. Its ability to generate drug-like compounds from 3D conformations is prominent due to its capability to bypass Open Babel post-processing and integrate bond details and molecular information.

**Results:**

For the gold standard QM9 dataset, CoDNet outperforms existing state-of-the-art methods with a validity rate of 99.02%. This competitive performance underscores the precision and efficacy of CoDNet’s drug design, establishing it as a significant advancement with great potential for enhancing drug development initiatives.

**Availability and implementation:**

https://github.com/CoDNet1/EDM_Custom.

## 1 Introduction

**Figure 1.**
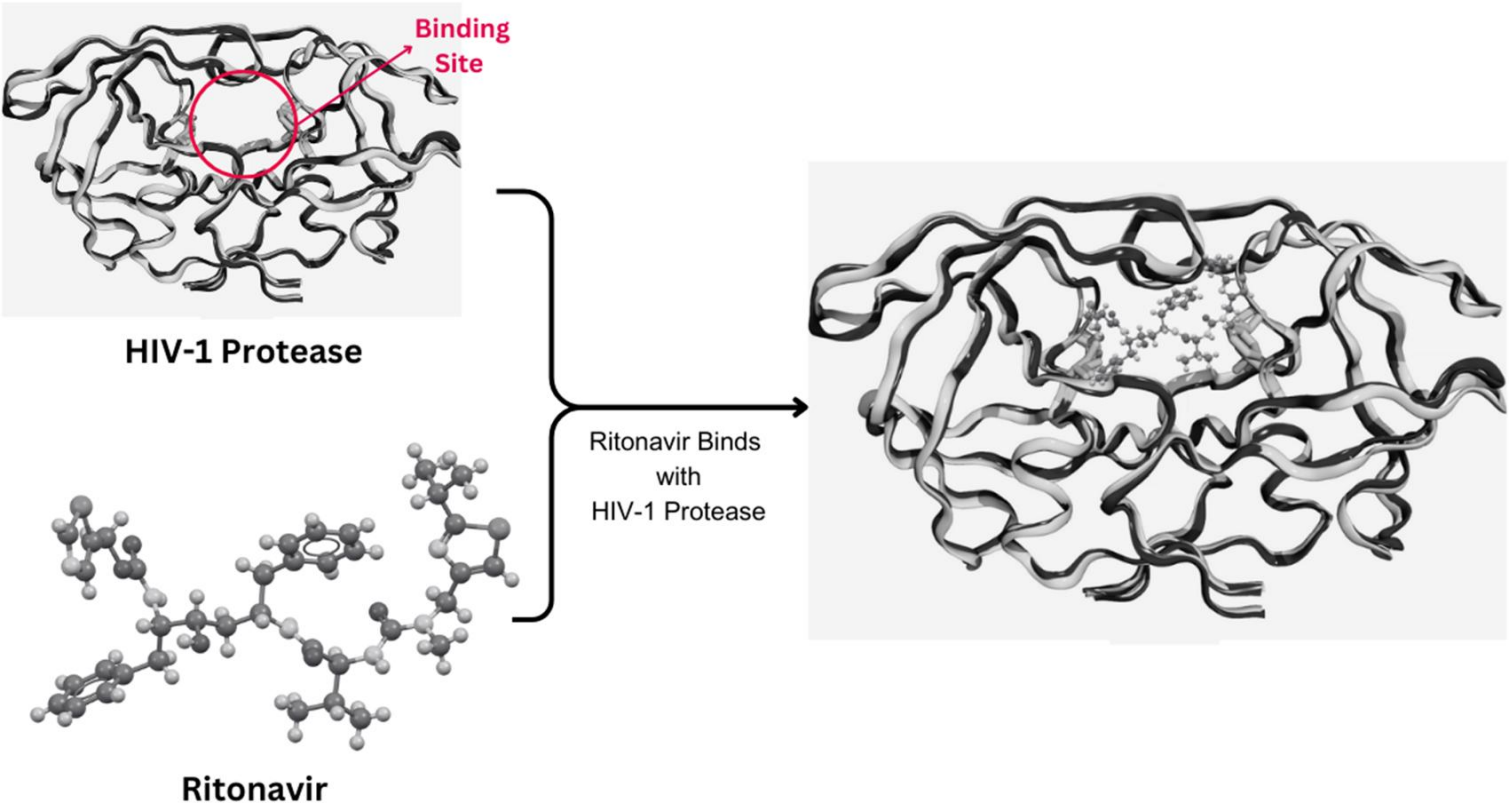
Interaction of HIV-1 protease with ritonavir, a structural insight into enzyme inhibition.

In the field of drug discovery, creating new drug-like compounds that can interact with therapeutic targets is crucial. For successful drug development, it is indispensable to have information about the therapeutic target, typically a disease-associated protein, as well as active ligands or molecules capable of effectively interacting with the target (Paul *et al.* 2021). For example, HIV-1 protease is a critical enzyme involved in the life cycle of the HIV virus, making it an essential therapeutic target. The three-dimensional (3D) structure of this protease provided valuable insights into its active site, which is key to its function. By leveraging structure-based drug design (SBDD), researchers developed drugs such as Ritonavir and Saquinavir illustrated in Fig. 1. These molecules are designed to specifically bind to and inhibit the protease, effectively blocking the enzyme’s activity, and preventing the virus from replicating. This approach has been pivotal in the development of effective antiretroviral therapies for managing HIV. Beyond HIV, a wide range of proteins and nucleic acids have been explored as therapeutic targets across various biomedical and pharmaceutical research domains. Bioinformatics and computational tools, such as molecular modeling and computer-aided drug design, play a significant role in identifying and evaluating these targets. Databases like the Therapeutic Target Database provide comprehensive information about therapeutic targets, associated diseases, pathways, and drugs/ligands, thereby accelerating research and innovation in drug discovery. Cross-linking these databases to sequence, 3D structure, and functional data further enhances the ability to design targeted therapies with precision (Chen *et al.* 2002).

Traditional approaches involved identifying lead compounds through high-throughput screening, where a large number of compounds with particular biological activities are systematically tested in laboratory settings (Macarron *et al.* 2011, Blay *et al.* 2020). However, this method is time-consuming and expensive (Cheng *et al.* 2012), with the entire drug discovery cycle taking up to 14 years and costing ∼2.8 billion dollars (Wieder *et al.* 2020, Walters and Barzilay 2021). Recent empirical evidence indicates a decline in new drugs entering the market attributed to the shortcomings encountered during clinical trials. A study in November 2018 estimated that the median cost of efficacy trials in 2015–16 was $19 million (Moore *et al.* 2018). Therefore, it is imperative to come up with a way to reduce the cost associated with the clinical trials and efficacy tests. The ability to design ligands that are predicted to have high efficacy and binding affinity to the target protein with the help of computational methods would serve as an effective way to ensure that fewer designs need to be lab tested and thus, reduce the cost of clinical trials and efficacy tests (Askr *et al.* 2023).

SBDD, in contrast to traditional methods, takes the rational approach of first identifying promising target proteins. Once the target protein is identified, it is extracted, purified, and its 3D structure is determined using experimental methods such as X-ray crystallography or NMR (Kneller *et al.* 2020). The availability of 3D structures of therapeutically important proteins facilitates the identification of binding cavities, forming the basis for SBDD. SBDD stands out as a precise and effective method for uncovering and refining lead compounds. This is achieved by focusing on the 3D structure of the target protein. SBDD involves the virtual screening of large chemical databases using different computational methods. Recently, deep surrogate docking models have been very popular when it comes to finding lead compounds (Clyde *et al.* 2021).

Recent advancements in geometric deep learning for modeling geometric structures of biomolecules, as highlighted by Isert *et al.* (2023), show a promising avenue for structure-based drug discovery. It plays a crucial role by enabling neural networks to learn non-Euclidean data like 3D molecular graphs and manifold data. Geometric deep learning methods for 3D SBDD utilize molecular representations such as 3D surfaces, grids, and graphs, which encode 3D coordinates or position matrices to learn structural information and construct molecular topologies during model training (Bai *et al.* 2024). Despite the significant advances in the application of deep learning, the creation of molecules that bind to target proteins remains an open challenge. Several neural network architectures have been proposed for molecule generation. Ragoza *et al.* (2022) utilized variational autoencoder on atomic density grid representation of the protein–ligand structures to generate new compounds. However, converting the output of atomic density grid into a molecular structure requires additional atom fitting and bond inference steps. Further studies overcame this issue by representing molecules as 3D graphs with atom positions and types, eliminating the need for extra post-processing steps. While Li *et al.* (2021) employed an autoregressive model specifically conditioned on the binding site. This method was further improved by Pocket2Mol (Peng *et al.* 2022) which utilize an E(3)-equivariant graph neural network that leverage rotational and translational symmetries in 3D space. While Pocket2Mol can detect rotational and translational changes, it is susceptible to accommodate such changes when the molecule is viewed from a different angle. This issue has been addressed by the incorporation of angles, with models that are using autoregressive approaches to generate atoms sequentially (Joshi *et al.* 2021, Liu *et al.* 2022). In recent research, advanced techniques like Feng *et al.* (2024) have introduced language model approaches for generating 3D molecules directly within protein binding pockets, offering a novel perspective on molecular generation. Similarly, equivariant diffusion models have shown its potential in molecule generation. MDM: Molecular Diffusion Model (Huang *et al.* 2023) suggests employing the diffusion model to create molecules, effectively addressing inter-atomic relationships and limited exploration. Complementing these approaches, Zhu *et al.* (2023) proposed a pharmacophore-guided deep learning method for generating bioactive molecules, further expanding the toolkit for computational molecular design. Extending these methodologies, DiffSBDD (Nakata *et al.* 2023), generates 3D molecular structures that are invariant to rotational and translational symmetries. Another equivariant diffusion model proposed conditioned the model using a selective iterative latent variable refinement (SILVR) variable that controls the extent to which the model is conditioned (Runcie and Mey 2023).

However, despite the efficacy of these models in generating chemically diverse molecules, none of them integrate both the 3D conformation and molecular graph of molecules. The molecular graph provides insight into chemical bonds and facilitates the identification of functional groups within a compound. On the other hand, the 3D conformation is crucial for determining the overall molecular shape and optimizing binding to protein binding sites. Existing models often neglect essential bond information by relying solely on 3D conformation for prediction, limiting their capacity for seamless differentiability throughout the entire process.

To address these gaps, we present CoDNet, an innovative diffusion model that merges molecular graphs and 3D conformations. By depicting molecules within a 3D graph, CoDNet concurrently generates 3D coordinates and bond details, ensuring a thorough portrayal of molecular structures and bolstering graph stability. Also by utilizing the Control Block with the base EDM (Vignac *et al.* 2023) to enable conditioning during the generation process, CoDNet ensures precise control over molecular design. When utilizing the pre-processed QM9 dataset, encompassing 134 000 organic molecules and represented by SMILES, XYZ, and InChI notations, our model excels in molecular generation, achieving a validation rate of 99.02% and a 99.902% score for connected components. Not only that, this model successfully deals with the optimization of the generated molecular structures. Even though many models depend on Open Babel for generating stabilized molecules, Open Babel does not work well on datasets that contain highly complex molecular structures, resulting in producing structures with invalid atoms or unstable bonds. But CoDNet addresses these complexities directly in the generation process, resulting in the generation of valid and stable molecular structures. With a novelty rate of 73% and a uniqueness score of 99.51%, CoDNet explores new chemical space and produces diverse molecular structures, showcasing its pivotal role in advancing structure-based drug discovery.

## 2 Materials and methods

### 2.1 CoDNet overview

**Figure 2.**
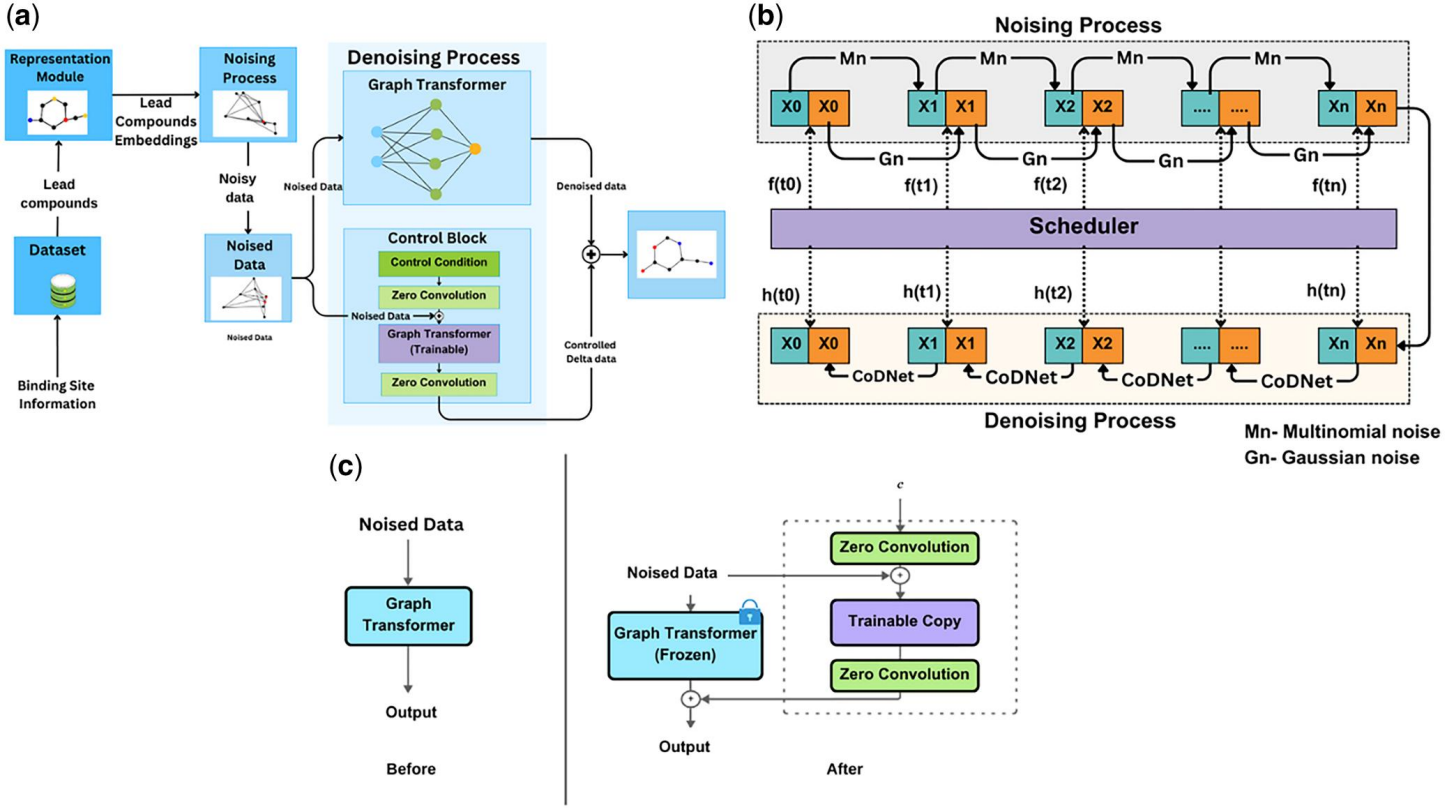
Complete workflow of the CoDNet. (a) The inference process of our model. Structural information of the binding site is inputted, and a cheminformatics database is searched for lead compounds. These compounds are converted into embeddings, through a noising process which is described in more details in (b), and passed into the Controlled EDM to generate the ligand. (b) The diffusion process of CoDNet for generating new molecules. Starting from a latent vector (“X0”), noise (“Mn” for Multinomial and “Gn” for Gaussian) is added, distorting the original molecular representation. Through iterative denoising steps using an rEGNN, the noisy vector is corrected closer to the original. The “Scheduler” guides the denoising, adjusting noise levels and injecting target molecule information. This results in a recovered latent vector that closely resembles the original noise-free version. (c) The conditioning process. The condition is inputted through the first zero convolution layer. The output is combined with a noise sample and passed into the trainable model. The trainable model’s output then goes through a second zero convolution layer. This output is concatenated with the frozen model’s output to produce the final conditioned output.

CoDNet takes a novel approach to the drug generation process. As depicted in the diagram in Fig. 2a, the model requires the structural details of the target’s binding site as input to begin the process. It then starts by searching for promising drug candidates, which are known as lead compounds. This is achieved by screening a large chemo-informatics database against said binding site to find the compounds that will bind to it. Once these lead compounds are identified, they are converted into machine-readable format before they are fed into the Controlled Equivariant Diffusion Model (EDM). The Controlled EDM then generates ligands with higher binding affinity to the target.

The Controlled EDM consists of an EDM (Vignac *et al.* 2023) which, as illustrated in Fig. 2b, iteratively introduces various types of noise, such as Multinomial Noise for atom types and bonds, and Gaussian Noise for 3D coordinate values. By introducing noise, the model is encouraged to explore a wider range of chemical space, leading to the generation of novel and diverse drug candidates. The output is then directed to a Graph Transformer that removes said noise. As the noise is gradually removed, the model refines its predictions and generates ligands that closely match the desired properties. The EDM can only generate unconditioned ligands. To incorporate the conditioning, a control block, illustrated in Fig. 2c is added.

### 2.2 Dataset preparation and pre-processing

CoDNet utilizes the QM9 dataset, which is a well-established benchmark in the field of drug discovery, providing a diverse range of molecular compounds (Yu *et al.* 2023). It comprises ∼134 000 organic molecules composed of H, C, N, O, and F, with a maximum of nine non-hydrogen atoms (Wu *et al.* 2018). The dataset includes 15 properties, such as geometric, energetic, electronic, and thermodynamic characteristics. The molecule geometries are optimized using the DFT-B3LYP/6-31G(2df, p) level of theory, and the dataset provides SMILES, XYZ, and InChI representations for each molecule (Ramakrishnan *et al.* 2014). Our analysis take advantage of the QM9 dataset pre-processed by Faber *et al.* (2017), which excluded 3000 molecules failing the InChI consistency check (Ramakrishnan *et al.* 2014). Additionally, 612 molecules unreadable by the RDKit Python package were removed. After these exclusions, the dataset was reduced to 130 217 molecules.

For further experimentation, we divided the dataset into 70% for training, 20% for validation, and 10% for testing.

### 2.3 Noise model for adding noise to both discrete and continuous data

In CoDNet, there are two types of data: discrete and continuous. The continuous data involves the coordinates of 3D conformers, while the discrete data comprises bond and charge information, as well as atomic properties of the molecular graph. To introduce noise to the coordinates of the 3D conformers, CoDNet utilizes a Gaussian distribution. However, before doing so, these positions are transformed to the zero center of mass subspace, ensuring that the total sum of added noise is zero and maintaining 3D equivariance in the model.

Regarding atomic properties, a categorical distribution is utilized, specifically incorporating noise through the marginal distribution within the categorical distribution (Vignac *et al.* 2023). The Equation (1) shows the noise model which is given by:


(1) $$q\left(G^{t}|G^{t-1}\right)∼N^{CoM}\left(\alpha^{t}R^{t-1},{\left(\sigma^{t}\right)}^{2}I\right)\times C\left(X^{t-1}Q_{x}^{t}\right)\times C\left(C^{t-1}Q_{c}^{t}\right)\times C\left(Y^{t-1}Q_{y}^{t}\right)$$

Here:

-
$q\left(G^{t}|G^{t-1}\right)$ : The translation property from state $G^{t-1}$ to state $G^{t}$.
-
$N^{CoM}\left(\alpha^{t}R^{t-1},{\left(\sigma^{t}\right)}^{2}I\right)$ : Gaussian distribution in the zero center-of-mass subspace, with mean $\alpha^{t}R^{t-1}$ and covariance ${\left(\sigma^{t}\right)}^{2}I$. Here, $\alpha^{t}$ is a scaling factor, $R^{t-1}$ represents the positions of the atoms in the previous state, $\sigma^{t}$is the standard deviation of the noise, and I is an identity matrix.
-
$C\left(X^{t-1}Q_{x}^{t}\right)$ : Categorical distribution for atomic properties, where $X^{t-1}$ represents atomic property values at time *t*-1, and $Q_{x}^{t}$ is a transition matrix for these properties.
-
$C\left(C^{t-1}Q_{c}^{t}\right)$ : Categorical distribution for charge information, where $C^{t-1}$ represents charge values at time *t*-1, and $Q_{C}^{t}$ is the transition matrix.
-
$C\left(Y^{t-1}Q_{y}^{t}\right)$ : Categorical distribution for bond information, where $Y^{t-1}$ represents bond information at time *t*-1, and $Q_{y}^{t}$ is the transition matrix.

CoDNet uses Gaussian noise within zero center-of-mass(CoM) subspace of the molecule, $\left(\epsilon ∼N^{CoM}\left(\alpha^{t}R^{t-1},{\left(\sigma^{t}\right)}^{2}I\right)\right)$, to identify the positions that are needed to obtain a roto-translation equivariant architecture (Xu *et al.* 2022). This ensures that the noise is applied in such a way that it maintains the rotational and translational symmetry of the molecular structure. Specifically, the Gaussian distribution is followed by the noise on its linear subspace of $\left(3\left(n-1\right)\right)$ dimension, satisfying $\left(\sum_{i==1}^{n}\epsilon_{i}=0\right)$, where *n* is the number of atoms and $\epsilon_{i}$ is the noise added to the *i*-th atom.

The posterior is defined as a product as well when generating new samples, this is shown in Equation (2):

(2) $$p_{\theta }\left(G^{t-1}|G^{t}\right)=\prod_{1\leq i\leq n}p_{\theta }\left(r_{i}^{t-1}|G^{t}\right)\;\times p_{\theta }\left(x_{i}^{t-1}|G^{t}\right)p_{\theta }\left(c_{i}^{t-1}|G^{t}\right)\prod_{1\leq i,j\leq n}p_{\theta }\left(Y_{ij}^{t-1}|G^{t}\right)$$

Here:

-
$p_{\theta }\left(G^{t-1}|G^{t}\right)$ : Posterior probability of state $G^{t-1}$ given state $G^{t}$.
-
$p_{\theta }\left(r_{i}^{t-1}|G^{t}\right)$ : Probability of the coordinate $r_{i}^{t-1}$ of atom *i* given state $G^{t}$.
-
$p_{\theta }\left(x_{i}^{t-1}|G^{t}\right)$ : Probability of the atomic property $x_{i}^{t-1}$ of atom *i* given state $G^{t}$.
-
$p_{\theta }\left(c_{i}^{t-1}|G^{t}\right)$ : Probability of the charge $c_{i}^{t-1}$ of atom *i* given state $G^{t}$.
-
$p_{\theta }\left(Y_{ij}^{t-1}|G^{t}\right)$ : Probability of the bond $Y_{ij}^{t-1}$ between atoms *i* and *j* given state $G^{t}$.

By marginalizing over the network prediction each term can be calculated, as shown in Equation (3):

(3) $$p_{\theta }\left(x_{i}^{t-1}|G^{t}\right)=\int_{x_{i}}P_{\theta }\left(x_{i}^{t-1}|x_{i},G^{t}\right)dp_{\theta }\left(x_{i}|G^{t}\right)\;\;\;=\sum_{x\epsilon X}q\left(x_{i}^{t-1}|x_{i}=x,G^{t}\right)p_{\theta }^{X}\left(x_{i}=x\right)$$

Here:

-
$p_{\theta }\left(x_{i}^{t-1}|G^{t}\right)$ : Marginal probability of atomic property $x_{i}^{t-1}$ given state $G^{t}$.
-
$P_{\theta }\left(x_{i}^{t-1}|x_{i},G^{t}\right)$ : Conditional probability of $x_{i}^{t-1}$ given $x_{i}$ and $G^{t}$.
-
$dp_{\theta }\left(x_{i}|G^{t}\right)$ : Probability measure over $x_{i}$ given state $G^{t}$.
-
$\sum_{x\epsilon X}\;$ : Summation over all possible atomic property values *X*.
-
$q\left(x_{i}^{t-1}|x_{i}=x,G^{t}\right)$ : Transition probability of $x_{i}^{t-1}$ given $x_{i}=x$ and $G^{t}$.
-
$p_{\theta }^{X}\left(x_{i}=x\right)$ : Probability of $x_{i}$ being equal to x within the categorical distribution.

### 2.4 Adaptive noise scheduler

CoDNet employs a Gaussian distribution for continuous data and a categorical distribution for discrete data. However, it is important to highlight that these two distributions do not consistently introduce noise. This discrepancy can lead to one type of data corrupting a graph significantly within a specific time frame, while the other may have a comparatively lesser impact. To address this issue, CoDNet implements an adaptive noise scheduler. This scheduler allows it to adjust the rate at which noise is introduced, ensuring a more balanced and controlled approach. By adopting such a strategy, CoDNet enhances its learning capabilities and mitigates the potential biases introduced by unequal noise contributions from different data types (Vignac and Frossard 2022, Madhawa *et al.* 2019). Equation (4) shows how this adjusted noise is calculated:


(4) $$\overset{-}{\alpha^{t}}=\cos{\left(\frac{\pi }{2}\frac{{\left(t/T+s\right)}^{v}}{1+s}\right)}^{2}$$

where (v) can take the form $\left(v_{r}),\left(v_{x}\right),(v_{y}\right)$, and $\left(v_{c}\right)$ representing atomic coordinates, types, bonds, and changes, respectively.

Here:

-
$\overset{-}{\alpha^{t}}$ : The adjusted noise scheduling factor at time t.
-
t : The current time step.
-
T:  The total time duration.
-
s : A scaling factor that adjusts the rate of noise introduction.
-
v : A power parameter that can take different forms to represent various types of data:
-
$v_{r}$ : Represents atomic coordinates.
-
$v_{x}$ : Represents atomic types.
-
$v_{y}$ : Represents bond information.
-
$v_{c}$ : Represents charge information

By using this adaptive noise scheduler, CoDNet ensures that the introduction of noise is balanced across different types of data, thereby improving its learning process and reducing potential biases.

### 2.5 A denoising transformer for noise removal while maintaining equivariance

CoDNet trains a neural network capable of effectively eliminating all previously introduced noise. To ensure that the neural network does not provide varying representations for a single graph, this model must exhibit SE3 equivariance.

SE3 equivariance refers to a property in which a model’s behavior remains consistent under the symmetries associated with the 3D Euclidean group (SE3), which includes translations and rotations in 3D space. For the neural network to exhibit SE3 equivariance, it must satisfy certain conditions. Firstly, the noise model employed should be equivariant to the action of the transformation group $G:\forall g\epsilon G,q\left(g\cdot z_{t}|g\cdot x\right)=q\left(z_{t}|x\right))$, where g is an element of G and $z_{t}$ represents the noisy observation of the molecular state at time t.

Secondly, during inference, a prior distribution $q_{\infty }$ should be invariant to the group action, meaning $q_{\infty }\left(g\cdot z_{t}\right)=q_{\infty }\left(z_{T}\right)$, where $z_{T}$ is the target clean state. Additionally, the noise introduced during inference should be processed by an equivariant neural network to ensure that the transition probabilities satisfy $p_{\theta }\left(g\cdot x_{t-1}|g\cdot z_{t}\right)=p_{\theta }\left(z_{t-1}|z_{t}\right)$.

Finally, the neural network should be trained using a loss function that maintains equivariance, expressed as $l\left(p_{\theta }\left(g\cdot x_{t-1}|g\cdot z_{t}\right),g\cdot x\right)=l\left(p_{\theta }\left(x|z_{t}\right),x\right)$. This ensures that the network effectively eliminates noise while preserving SE3 equivariance in its representations of molecular structures.

To achieve this, CoDNet employs a graph transformer architecture, encompassing essential components such as self-attention blocks, normalization layers, feed-forward networks, and, notably, rEGNN (Relaxed Graph Neural Network) (Satorras *et al.* 2021). rEGNN is not limited to the coordinates data of the 3D conformers; it also incorporates nodal features, including atomic information, bond details, and edge information. This model incorporates external information through message-passing mechanisms. CoDNet uses the E3Norm normalization layer to ensure that data normalization aligns with our goal of achieving SE3 equivariance in the neural network (Liao and Smidt 2023). The rEGNN is implemented using Equation (5).


(5) $${\left[\Delta_{r}\right]}_{ij}=\mathrm{cat}\left({\left|\left|r_{i}-r_{j}\right|\right|}_{2},{\left|\left|r_{i}\right|\right|}_{2},{\left|\left|r_{j}\right|\right|}_{2},\cos\left(r_{i},r_{j}\right)\right)\;r_{i}\leftarrow r_{i}+\sum_{\;j}\phi_{m}\left(X_{i},X_{j},{\left[\Delta_{r}\right]}_{ij},Y_{ij}\right)\left(r_{j}-r_{i}\right)$$

Here:

-
${\left[\Delta_{r}\right]}_{ij}$ : The concatenation of features between atoms i and j.
-
$\cos\left(r_{i},r_{j}\right)$ : The cosine of the angle between $r_{i}$ and $r_{j}$.
-
$r_{i}$ : The coordinates of the i-th atom.
-
$\phi_{m}$ : The message-passing function.
-
$X_{i},X_{j}$ : Nodal features of atoms i and j.
-
$Y_{ij}$ : Edge features between atoms i and j.

For integration into a transformer architecture, transformers are found to be competent at stabilizing the self-attention mechanism over many layers. So to ensure SE(3)-equivariance, changes were made to the feed-forward neural network and normalization layers. Multilayer perceptrons (MLPs) are applied in parallel on each node and edge for the processing of each component done by the feed-forward neural network. As each coordinate cannot be treated separately, CoDNet applies the MLP using Equation (6).

(6) $$\mathrm{PosMLP}\left(R\right)={\prod \;}^{\mathrm{CoM}}\left(\mathrm{MLP}\left|\left|R\right|\right|\right)\frac{R}{\left|\left|R\right|\right|+\partial }\epsilon R^{n\times 3}$$

Here:

-
$\mathrm{PosMLP}\left(R\right)$ : The position-based MLP applied to the all coordinates R.
-
R : A matrix of coordinates.
-
$\left|\left|R\right|\right|$ : The Euclidean norm of R.
-
∂ : A small constant added for numerical stability.
-
$\epsilon R^{n\times 3}$ : Denotes that R is an *n* × 3*n* × 3 matrix, where *n* is the number of atoms, and each atom has 3 coordinates.

$\prod^{\mathrm{CoM}}\;$ is the projection of the coordinates on the linear subspace with center-of-mass at 0, as shown in Equation (7):

(7) $${\prod \;}^{\mathrm{CoM}}{\left(R\right)}_{i}=r_{i}-\frac{1}{n}\sum_{i=1}^{n}r_{i}$$

where:

- *R*: The matrix of coordinates
-
$r_{i}$ : position vector of the *i*-th atom
- *n*: Total number of atoms

The choice of the normalization layer also depends on the problem symmetries. While some graph transformer models use batch normalization, this layer is not equivariant, unlike layers like set normalization or layer normalization. For SE(3) equivariance, the normalization shown as mentioned in Equation (8) is used.


(8) $$E3\mathrm{Norm}\left(R\right)=\Gamma \frac{\left|\left|R\right|\right|}{\bar{n}+\partial }\frac{R}{\left|\left|R\right|\right|}=\gamma \frac{R}{\left|\left|R\right|\right|}\;\mathrm{with}\overset{-}{n}=\sqrt{\frac{1}{n}\sum_{i=1}^{n}{\left|\left|r_{i}\right|\right|}^{2}}$$

### 2.6 Control block to enable conditioning into the generation process

CoDNet unconditionally trains the transformer model so that it can generate a molecule design. Then, it creates a copy of the trained transformer model and freezes its parameters. In the copied model, it adds zero convolutions at the beginning and the end of its trainable parameters. The first zero convolution layer will be fed with an input layer representing the conditions. At the end of it, residual connections are added by incorporating a noise sample. Then, CoDNet fits this modified vector into the trainable model. The output from the trainable model goes through the second zero convolution layer before being concatenated to the frozen model’s output. This result is considered as the final output, $y_{c}$. The output of the complete conditioned model $y_{c}$ is represented in Equation (9). This entire process is illustrated in .(*c*).


(9) $$y_{c}=F\left(x;\theta \right)+Z\left(F\left(x+Z\left(c;\theta_{z1}\right);\theta_{c}\right);\theta_{z2}\right)$$

Here:

-
$y_{c}$ : The final conditioned output of the model.
-
F : The function representing the transformer model.
-
x : The input to the transformer model.
-
θ : The parameters of the original, unconditionally trained transformer model.
-
Z : The zero-convolution layer.
-
c : The conditions provided to the model
-
$\theta_{z1}$ : The parameters of the first zero convolution layer.
-
$\theta_{c}$ : The parameters of the trainable copied transformer model.
-
$\theta_{z2}$ : The parameters of the second zero-convolution layer.

The reason for using zero convolution at the beginning is to refrain CoDNet’s conditions from distorting the output of the model. This ensures that no additional loss is generated as a result of the conditions, while the existing loss remains minimal. Retaining this minimal loss ensures that the weights of the model start to change gradually. As the training progresses, the weights of the zero convolution normalize, conditioning the output of the model.

CoDNet adds noise to information about charges, atom type and bond type and returns the coordinates through a reverse process called the regression process. CoDNet uses mean squared error for the coordinates, and categorical cross-entropy for the discrete data like charges, atom type, bond type, etc. By summing up these losses, CoDNet tries to minimize the final loss.

### 2.7 Testing and evaluation

We have performed direct comparison of CoDNet with state-of-the-art models such as GSchNet, EDM, EDM+OBabel, MiDi (uniform), and MiDi (adaptive). Its performance is examined through a multi-pronged approach on the QM9 data set.

We have tested for CoDNet validity, connected component, novelty, uniqueness, valency, bond lengths, and angles following the method of Le *et al.* (2023).

1. *Validity*: To judge its ability to produce chemically valid compounds. The percentage of valid compounds generated out of all the compounds generated is calculated so the greater the value, the better the performance.
2. *Connected component*: To see how connected the compounds generated by it are in order to evaluate its ability to capture complex molecular relationships. The percentage of completely connected compounds generated out of all the compounds generated is calculated so the greater the value, the better the performance.
3. *Novelty*: To evaluate its ability to generate new drugs. The percentage of novel compounds generated out of all the compounds generated is calculated so the greater the value, the better the performance.
4. *Uniqueness*: To evaluate its ability to generate distinct molecular representation. The percentage of unique compounds generated out of all the compounds generated is calculated so the greater the value, the better the performance.
5. We have evaluated its ability to capture physical properties by calculating the valency, bond lengths, and angles between the atoms of the compounds generated by it.
6. *Valency*: The number of chemical bonds an atom can form, visualized as “arms” reaching out to connect with other atoms. The lower the value is, the better.
7. *Bond length*: The distance between the nuclei of two bonded atoms, typically measured in Angstroms (Å) and pictured as the space between the “arms” connecting the atoms. The lower the value is, the better.
8. *Angles*: Angles measure the space between bonds connected to a central atom, influencing molecular shape and stability. The ideal bond angle is 109.5° for tetrahedral geometry, although deviations may occur due to factors like lone pair repulsion, resulting in smaller angles (Kawa 1988).

## 3 Results

To assess the effectiveness of CoDNet, we conducted a comprehensive set of evaluations, employing diverse tests to measure its performance across various molecular attributes. These evaluations include tests for validity, connected components, novelty, uniqueness, valency, bond lengths, and bond angles. The selection of these specific metrics is rooted in their significance in determining the model’s ability to generate chemically valid, diverse, and stable molecular structures. The results of the evaluations for CoDNet are summarized in Table 1, providing an overview of its performance across different metrics.

**Table 1.** Summarization of the performance of CoDNet.

Matrix	Score (%)
Validity	99.02
Connected components	99.902
Novelty	73.00
Uniqueness	99.51
Valency	0.47
Bond length	0.12
Bond angles	2.33

These scores reflect CoDNet’s exceptional capability to generate valid, connected, novel, and unique molecules, while maintaining favorable valency and geometric characteristics.

**Figure 3.**
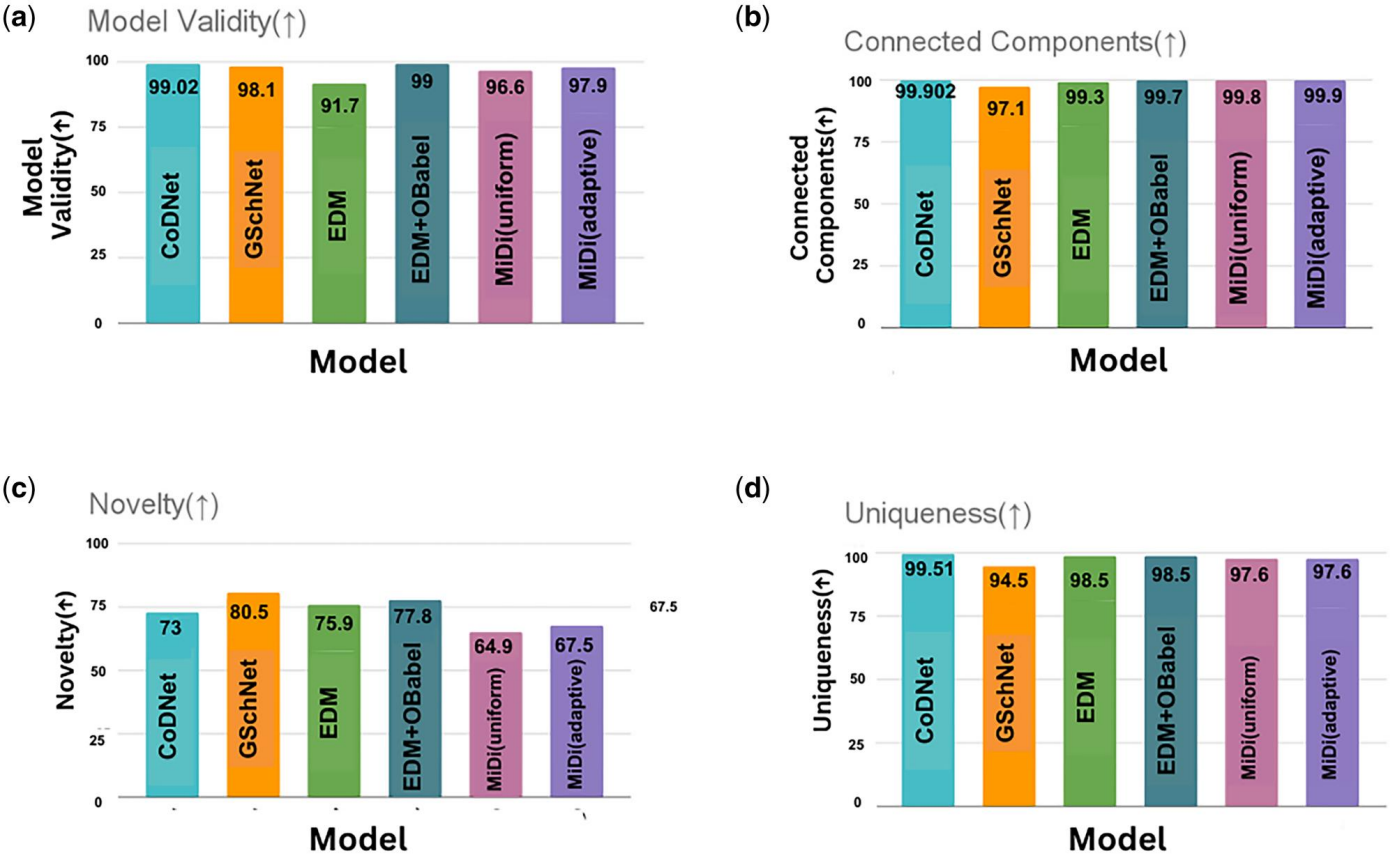
This figure represents a comprehensive comparative analysis of CoDNet against state-of-the-arts models based on key metrics, like (a) validity, (b) connected components, (c) novelty, and (d) uniqueness which outperformed existing state-of-the art methods.

In addition to evaluating the standalone performance of CoDNet, we conducted a comparative analysis against state-of-the-art models in this research domain. Figure 3 illustrates a comprehensive analysis of CoDNet against several other models, emphasizing its superior performance in terms of validity, connected components, novelty, and uniqueness. CoDNet emerges as the top performer in terms of validity, achieving a score of 99.02, signifying its exceptional ability to generate valid compounds. Following closely is GSchNet with a validity score of 98.1, while EDM trails behind at 91.7. CoDNet also excels in generating connected compounds, boasting a score of 99.902, outpacing competitors such as EDM, GSchNet, EDM+OBabel, and MiDi (uniform). In terms of novelty, CoDNet maintains a competitive edge with a score of 73, showcasing its capability to produce chemically unique compounds. While GSchNet, EDM+OBabel, and MiDi (adaptive) also demonstrate good novelty, CoDNet remains a standout performer. The uniqueness metric further emphasizes CoDNet’s excellence, with a score of 99.51, indicating a high degree of diversity in the generated compounds. In summary, CoDNet consistently outperforms other models across most evaluated attributes, making it a promising candidate for compound generation tasks. Although GSchNet, EDM, EDM+OBabel, and MiDi models display competitive performance, CoDNet demonstrates a notable superiority in overall quality and diversity.

**Figure 4.**
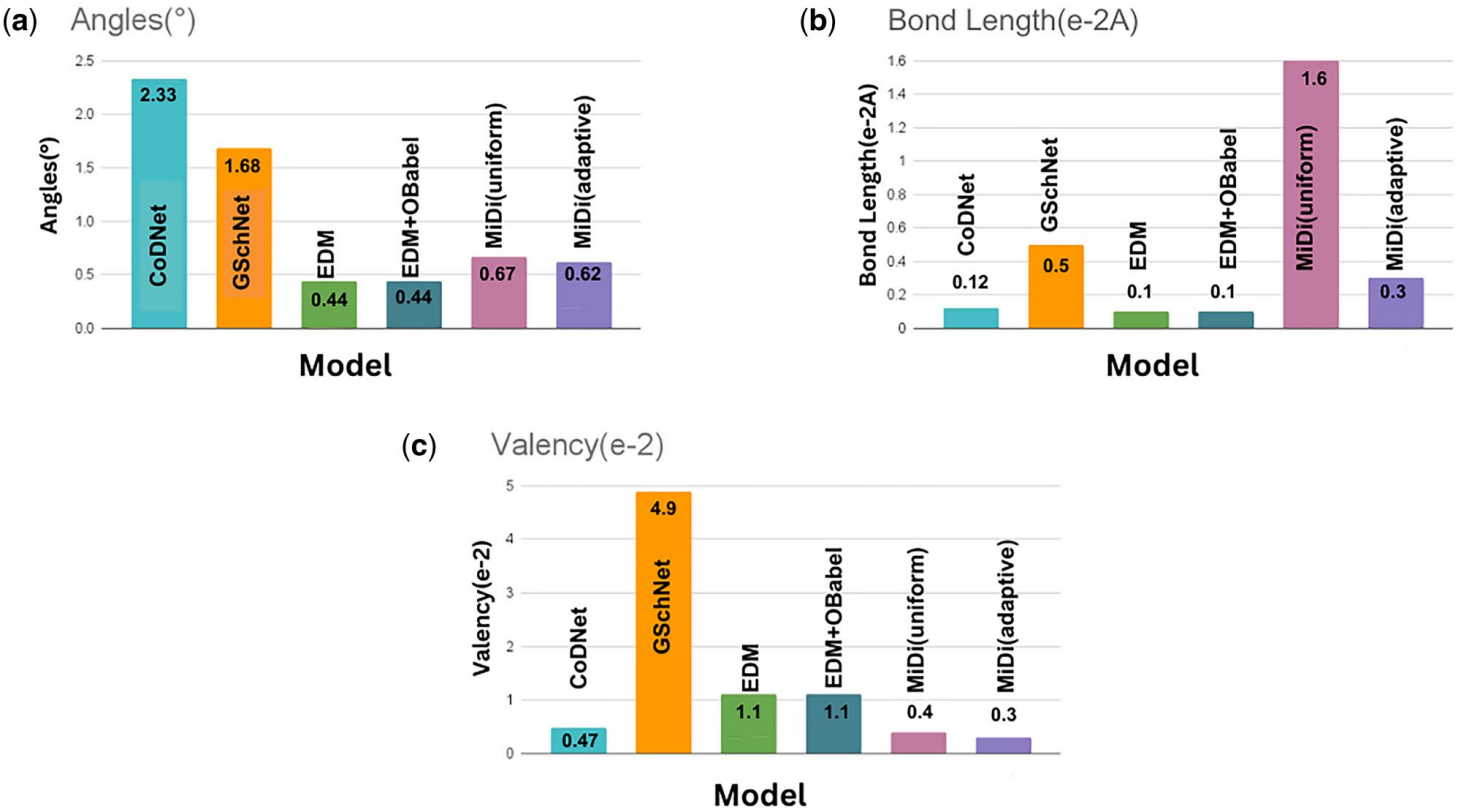
This figure provides a comprehensive comparison of CoDNet with various other models, assessing critical attributes such as (a) bond angles, (b) bond length and (c) valency.

Figure 4 provides a detailed comparison of CoDNet with various models, focusing on critical attributes such as valency, bond lengths, and bond angles. These visual representations further underscore CoDNet’s notable superiority over existing models, solidifying its position as a promising candidate for molecular generation tasks.

In terms of valency, CoDNet stands out with a notably lower value of 0.47, indicating a more efficient utilization of electron pairs compared to its counterparts. In contrast, GSchNet exhibits a higher valency at 4.9, suggesting potential challenges in managing electron pairs effectively. The models EDM, EDM+OBabel, MiDi (uniform), and MiDi (adaptive) showcase intermediate valency values, with CoDNet demonstrating superior performance in this regard. Examining bond lengths, CoDNet excels with the smallest bond lengths at 0.12, implying tighter and more stable molecular structures. GSchNet, EDM, and EDM+OBabel, however, display larger bond lengths at 0.5, 0.1, and 0.1, respectively, indicating potential structural differences and deviations from optimal bond distances. Although MiDi (uniform) and MiDi (adaptive) exhibit variations in bond lengths, CoDNet remains notably superior in achieving compact molecular configurations. Analyzing bond angles, CoDNet demonstrates a favorable angle of 2.33 degrees, indicating a more preferred geometric arrangement of atoms in the molecular structure. GSchNet and MiDi (uniform) display higher bond angles at 1.68 and 0.67, respectively, potentially leading to less stable molecular conformations. Conversely, EDM, EDM+OBabel, and MiDi (adaptive) present lower bond angles. Overall, CoDNet outperforms the compared models across all evaluated attributes, showcasing lower valency, smaller bond lengths, and favorable bond angles. These results suggest that CoDNet excels in efficiently representing molecular structures with improved stability and geometric accuracy compared to its counterparts.

## 4 Discussion

The assessment of CoDNet underscores its substantial progress in conditional molecule generation, setting itself apart by concurrently generating both graph structures and 3D coordinates. This unique capability distinguishes CoDNet from existing models that predominantly focus on 3D conformations. The extensive evaluation on QM9 datasets encompasses a diverse set of metrics, offering a comprehensive analysis of its efficacy.

Chemical validity is of utmost importance, and CoDNet excels in this regard, surpassing counterparts. Additionally, CoDNet demonstrates exceptional performance in generating structurally coherent molecules, showcasing its proficiency in the essential metric of connected components.

According to Vignac and Frossard (2022), QM9 represents a complete set of molecules that adhere to a specific set of predefined constraints. In this context, “novel” molecules simply fall outside the dataset’s rigid constraint. Novelty assessment is a critical facet of evaluating molecule generation models, and CoDNet generates novel structures by integrating a control network with the diffusion process, allowing for greater control over constraints. A substantial portion of these structures is absent from the training dataset, highlighting CoDNet’s prowess in exploring uncharted chemical spaces.

CoDNet exhibiting a higher uniqueness than all its counterparts is particularly noteworthy. This attribute holds particular significance in drug discovery and materials science, where a diverse set of molecules enhances the likelihood of identifying compounds with specific desirable properties.

Geometric considerations further contribute to the model’s prowess. CoDNet exhibits precision in maintaining realistic valency, reflected in a notably low valency value. Lower valency values indicate that atoms form the correct number of bonds, crucial for stability and chemical feasibility. The evaluation extends to bond lengths and angles, with CoDNet demonstrating proficiency in reproducing realistic and accurate values.

**Figure 5.**
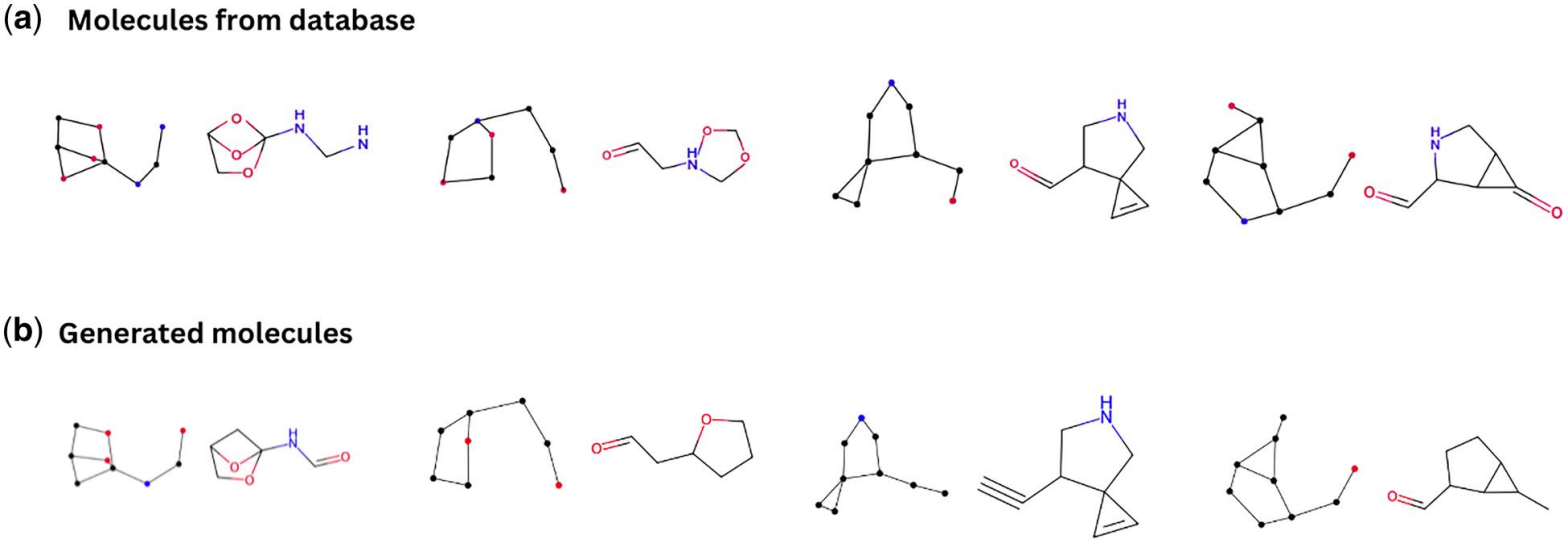
The molecules shown on (a) are sampled from the database, while the molecules shown on (b) were generated by CoDNet. The molecules that are produced demonstrate a significant level of resemblance to their database counterparts, which validates the stability of CoDNet.

Additionally, CoDNet demonstrates a profound understanding of drug-like molecules, as illustrated in Fig. 5. The molecules in Fig. 5a are from the database, while those in Fig. 5b were generated by CoDNet. The molecules produced demonstrate a significant level of resemblance to their database counterparts, validating the stability and applicability of our model in generating molecules with desirable drug-like features. The binding affinity of a ligand to a target protein is largely determined by the ligand’s structure and its functional groups. Given that the molecules generated by our model closely resemble actual drug molecules from the database, they are highly likely to bind effectively to the target’s binding site. As we have used a graph-based representation for molecular structures, where vertices represent atoms and edges represent chemical bonds. The generated structures comply with basic chemical constraints (e.g. valency, bond length, and angles). The novelty and uniqueness metrics confirm that these structures are innovative compared to known molecules. While these are preliminary results, they provide a strong foundation for downstream validation steps such as energy minimization and biological activity testing.

In the context of drug–target interaction prediction, these molecular structures are well-suited for both molecular docking simulation (MDS)-based approaches and machine learning (ML)-based methods. The MDS approach can analyze how these molecules bind to biological targets, while ML-based methods can leverage structural data to predict interactions efficiently. These computational methods provide a high-efficiency and low-cost alternative to traditional *in vitro* experimental techniques (Zhao *et al.* 2023).

Graphical structures follow the basic rules of chemistry, such as correct valency, realistic bond lengths, and bond angles, ensuring they are chemically valid and stable. They are also fully connected, confirming that they represent single molecules and not fragmented systems. From a chemical perspective, the molecules are stable, likely synthesizable, and offer novel scaffolds that explore new chemical spaces. This novelty could lead to breakthroughs, as these structures are different from what’s already known.

From a biological point of view, the molecules show potential to interact with biological targets due to their diverse shapes and connectivity. The use of *in silico* computational techniques such as docking studies and the addition of functional groups can further optimize their biological activity and improve properties like solubility and absorption. These methods provide a robust framework for enhancing drug discovery pipelines.

These initial results, grounded in chemical and biological reasoning and supported by computational predictions, highlight the potential of our generated molecules as promising candidates for further drug development.

In summary, CoDNet emerges as a leading model in unconditional molecule generation, showcasing exceptional performance in generating chemically valid and stable molecular structures. The presented results not only establish its superiority over existing models but also emphasize its potential to significantly impact drug discovery. The model’s adeptness in generating diverse, valid and novel molecular structures marks a substantial stride in advancing the field of drug discovery and computational chemistry.

## Supplementary Material

vbaf031_Supplementary_Data

## Data Availability

None declared.
