# Supplementary material for: CoDNet: controlled diffusion network for structure-based drug design
Source: Bioinform Adv. 2025 Feb 19;5(1):vbaf031. doi: 10.1093/bioadv/vbaf031 (PMC11886848; doi:10.1093/bioadv/vbaf031)
Supplement: vbaf031_Supplementary_Data [file vbaf031_supplementary_data.zip › Supplementary Materials for Online.docx]

**Supplementary Document**

**CoDNet: Controlled Diffusion Network for Structure Based Drug Design**

Fahmi Kazi Md.^1^, Shahil Yasar Haque^1^, Eashrat Jahan^1^, Latin Chakma^1^, Tamanna Shermin^1^, Asif Uddin Ahmed^1^, Salekul Islam^2^, Swakkhar Shatabda^3^, Riasat Azim^1,*^

^1^Department of Computer Science and Engineering, United International University, Dhaka 1212, Bangladesh, ^2^Department of Electrical Computer and Engineering, North South University, Dhaka 1229, Bangladesh, ^3^Department of Computer Science and Engineering, Brac University, Dhaka 1212, Bangladesh.

* Riasat Azim. Tel: +8801796619200; Email: riasat@cse.uiu.ac.bd

**PRELIMINARIES**

**Target:**

In pharmacology and medicine, a 'target', shown in Figure 1, refers to a specific molecule, receptor, enzyme, or other key biological element in the body associated with a disease. Drugs are designed to interact with these targets to achieve a desired therapeutic effect. When a drug interacts with its designated target, it can either enhance or inhibit the target's activity, leading to the intended pharmacological response. This response can include disease treatment, symptom relief, or the facilitation of specific biological processes.


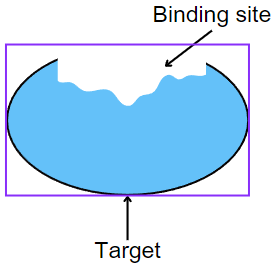


Figure 1: Target and Binding site

**Binding Site:**

A binding site is like a docking spot on a molecule, such as a protein or enzyme, where a drug or ligand can attach, as shown in Figure 1. Think of it as a key fitting into a lock. The drug is carefully shaped to match the binding site's size, shape, and chemical properties, ensuring a strong bond. When the drug links with the binding site, it can influence the molecule's function, either enhancing it or inhibiting it. Several computational approaches have proven to be quite successful in identifying the binding site.

**Lead compounds:**

Lead compounds, shown in Figure 2, serve as foundational building blocks in the early stages of drug discovery. They are selected for their initial positive effects and their ability to bind to the target. They form the groundwork for developing improved drugs. Scientists focus on refining the structure of lead compounds to enhance their efficacy and precision. These lead compounds serve as the starting point for the development of new medicines that not only work effectively but are also safe for use. Recently, deep surrogate docking models have been very popular when it comes to finding lead compounds.


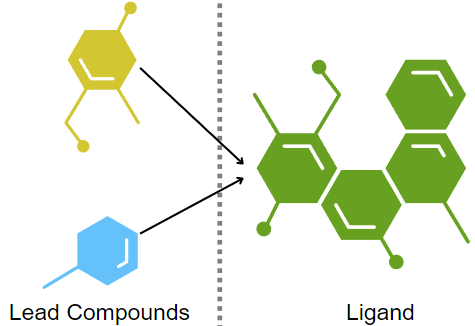


Figure 2: Lead Compounds and Ligand

**Ligands:**

Ligands, shown in Figure 2, are large molecules crafted by refining the structure of lead compounds during the search for new drugs. They are designed to specifically attach to target bio-molecules and influence their function. Think of ligands as keys fitting into locks, triggering chemical reactions that produce beneficial effects. They can also direct signal pathways in cells and regulate bodily processes.

**S.M.I.L.E:**

A way to show how drugs are built is using the simplified molecular input line entry system (SMILE). It follows certain rules to describe how molecules are shaped in a textual way. As shown in Figure 3, S.M.I.L.E. uses letters to represent atoms and special symbols for bonds and features like aromatic shapes or cycles.


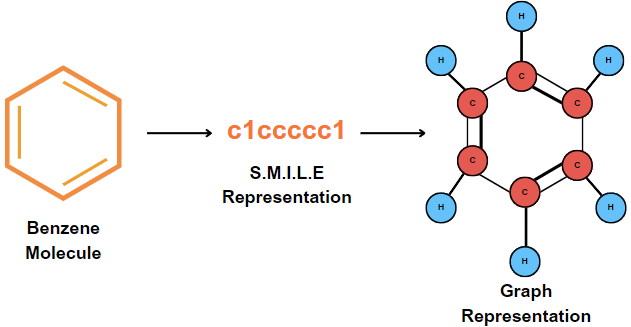


Figure 3: S.M.I.L.E. Representation and Graph Representation

**Graphical Representation:**

It is good at figuring out how proteins do their job and interact with other stuff. as shown in Figure 3, each atom is represented with a dot, and the lines between them represent the bond between them, showing how close or far they are from each other and at what angles they're arranged. This way of showing things works better in terms of size when compared to the 3D shapes used in convolutional neural networks.

**Transformer:**

The transformer architecture is designed to work with embeddings of a fixed size, no matter where the input data comes from. In a transformer model, the input data is transformed into these embeddings, which are like compact representations in the form of vectors that capture the essence of the data. These embeddings ensure that the model can process and understand the data consistently, regardless of its diverse sources.

**Denoising Diffusion Models:**

Diffusion models are generative models, they use data to generate more data. Diffusion works by learning the underlying probability distribution of a dataset through a denoising process. Diffusion models are trained through a progressive process of adding noise making it noisier at each step [1]. Afterward, the noisy data is then cleaned to recover the original data through a reversed diffusion process.

The forward processing can be represented by the equation 1:

| $q\left( z_{1},\ldots,z_{T} \vert x \right)=q\left( z_{1} \vert x \right)\prod_{t=1}^{T} q\left( z_{t} \vert z_{t-1} \right)$ | 1 |
| --- | --- |

In this equation $(q)$ is the noise model which takes data point $(x)$ as input and generates progressively noisy data points $(\left( z_{1},\ldots,z_{T} \right))$ over $(T)$ timesteps. This equation follows the Markovian chain rule.

The reverse processing can be represented by the equation 2:

| $p_{\theta}\left( z_{t-1} \vert z_{t} \right)=\int_{x} p_{\theta}\left( z_{t-1} \vert x,z_{t} \right) dp_{\theta}\left( x \vert z_{t} \right)$ | 2 |
| --- | --- |

$(p_{\theta})$ takes the noisy data $(z_{t})$ as input and then it reverses the diffusion trajectory. This equation shows how denoising works during data generation. Through these processes of adding and removing noise, the diffusion model generates newer data by passing randomly sampled noise through the reverse processing equation [1].

Even though equation (2) provides efficient training, it still faces problems as it requires efficient computation of $(p_{\theta}\left( z_{t-1} | x,z_{t} \right))$ and the integral, which is not always possible. To keep equation \eqref{eq:forward} easy to deal with, two frameworks can be used. Gaussian noise for continuous data, and discrete state-space diffusion for categorical data.

$(q\left( z_{t} | z_{t-1} \right)\sim\mathcal{N}\left( \alpha_{t}zt,\sigma_{t}^{2}I \right))$defines the Gaussian diffusion processes, here $(\left( \alpha_{t} \right)t\leq T)$ determines the retained signal and $(\left( \sigma_{t} \right)t\leq T)$ determines the amount of noise to be added [1]. The normal distributions are stable under composition, we can express $(q\left( z_{t} | z_{t-1} \right)\sim\mathcal{N}\left( \alpha_{t}zt,\sigma_{t}^{2}I \right))$ with $(\bar{\alpha_{t}}=\prod_{s=1}^{t} \alpha_{s})$ and$(\bar{\sigma_{t}^{2}}=\sigma_{t}^{2}-\alpha_{t}^{2})$. So, this satisfies

$$q\left( z_{t} | z_{t-1} \right)\sim\mathcal{N}\left( \mu_{t}x+v_{t}z_{t},\bar{\sigma_{t}^{2}}I \right),$$

with

$\mu_{t}=\bar{\alpha_{s}}\left( 1-\alpha_{t}^{2}\bar{\sigma_{t}^{2}} \right),v_{t}=\left( \alpha_{t}\bar{\sigma_{t-1}^{2}}/\bar{\sigma_{t}^{2}} \right)$;

and;

$$\bar{{\sigma_{t}}_{t}}=\bar{\sigma_{t}^{2}}\left( 1-\alpha_{t}^{2}\bar{\sigma_{t-1}^{2}}/\bar{\sigma_{t}^{2}} \right)$$

Discrete diffusion considers that $(x)$ belongs to one of $(d)$ classes. To represent the probability of jumping from one class to another at each step the $(d)x(d)$ size square matrices $(\left( Q_{1},\ldots,Q_{T} \right))$ can be used. The noise model for the next state $(z_{t})$ given the previous state $(z_{t-1})$ is a categorical distribution over $(d)$ possible classes that read as$(q\left( z_{t} | z_{t-1} \right)\mathcal{\sim C}\left( z_{t-1} \right)Q_{t})$. The process is Markovian; hence we simply use $(q\left( z_{t}=j | x \right)=\left[ x\bar{Q^{t}} \right]_{j})$ with $(\bar{Q^{t}}=Q^{1}Q^{2}\ldots Q^{t})$ [1]. $(q\left( z_{t} | z_{t-1}) \right)$ can also be computed in closed form using the Bayes rule and the Markovian property. $(\odot)$ denotes pointwise product and $(Q^{'})$ is transpose of$(Q)$, so it can be represented as in equation 3

| $q\left( z_{t}=j \vert z^{t},x \right)\infty z^{t}\left( Q^{t} \right)^{'}\odot x\bar{Q^{t-1}}$ | 3 |
| --- | --- |

**SE(3)-Equivariance With Diffusion Models:**

Molecules can undergo translations and rotations, but the arrangements of their molecules do not have a predetermined order. To ensure the effectiveness of the model, the models must be equivariant to the symmetries associated with molecules. Diffusion models can achieve equivariance to a transformation group $(\mathcal{G})$ through various conditions. First is the noise model must be equivariant to the action of $(\mathcal{G}):(\forall g\epsilon\mathcal{G},q\left( g\cdot z_{t} | g\cdot x \right)=q\left( z_{t} | x \right))$. Second is using prior distribution $(q_{\infty})$ at inference to the group action to be invariant, i.e., $(q_{\infty}\left( g\cdot z_{t} \right)=q_{\infty}\left( z_{T} \right))$, and this noise should be processed by an equivariant neural network in order to ensure that$(p_{\theta}\left( g\cdot x_{t-1} | g\cdot z_{t} \right)=p_{\theta}\left( z_{t-1} | z_{t} \right))$. Lastly, the network should be trained on a loss function that satisfies $(l\left( p_{\theta}\left( g\cdot x_{t-1} | g\cdot z_{t} \right),g\cdot x \right)=l\left( p_{\theta}\left( x | z_{t} \right),x \right))$.

Many generative models for molecules use EGNN layers as these are much more affordable, compared to the various other architectures for denoising that are much more computationally expensive due to the manipulation of spherical harmonics. At a high level, EGNN updates $(\left( r_{i} \right))$ coordinates of a graph recursively with node features $(\left( x_{i} \right))$ and edge features $(\left( y_{\mathrm{ij}} \right))$ using equation 4

| $r_{i}\leftarrow r_{i}+\sum_{j} c_{ij}m\left( \left\vert\left\vert r_{i}-r_{j} \right\vert\right\vert,x_{i},x_{j},y_{ij} \right)\left( r_{j}-r_{i} \right)$ | 4 |
| --- | --- |

So the primary feature of this parameterization is that function \(m\) only takes rotation-invariant arguments. Combined with linear terms in $(r_{j}-r_{i})$, ensures that the network is rotation-equivariant. Finally, we note that term $(c_{\mathrm{ij}}=\left| \left| r_{i}-r_{j} \right| \right|+1)$ is necessary for numerical stability when concatenating many layers [1].

**GAP ANALYSIS**

| Paper Title | Main Focus | Gap/Opportunity Identified |
| --- | --- | --- |
| Generating 3D molecules conditional on receptor binding sites with deep generative models by Ragoza et al 2022. | Conditional generation of molecules based on receptor binding sites. | Increasing the accuracy with which compounds with specific binding affinities and characteristics for receptor sites are generated. |
| Structure-based de novo Drug Design using 3D Deep Generative Models by Li et al 2021. | Utilizing 3D generative models for drug design. | Improving the production of novel drug-like compounds with improved characteristics and conformations. |
| Pocket2Mol: Efficient Molecular Sampling Based on 3D Protein Pockets by Peng et al 2022. | Efficient molecular sampling from protein pockets. | Generating compounds that interact positively with protein binding sites using more efficient methods. |
| Structure-aware Generation of Drug-like Molecules by Droter et al 2020. | Incorporating structural information for drug-like molecule generation. | Improving the strategy to take into account various chemical structures while maintaining drug-likeness and synthesizability. |
| Generating 3D Molecules for Target Protein Binding by Liu et al 2022. | Generating molecules for specific protein binding. | Overcoming difficulties with generating compounds with suitable pharmacological characteristics in addition to their ability to bind to target proteins. |
| MolGenSurvey: A Systematic Survey in ML Models for Molecule Design by Du et al 2022. | Survey of machine learning models for molecule design. | Identifying problems in existing machine learning techniques and proposing possible areas for improvement. |
| MDM: Molecular Diffusion Model for 3D Molecule Generation by Hoogeboom et al 2022. | Molecular diffusion model for molecule generation. | Employing diffusion-based models to improve the accuracy as well as efficiency of molecule generation while resolving their drawbacks. |
| Structure-Based Drug Design With Equivariant Diffusion Models by Arne et al 2022. | Utilizing equivariant diffusion models for drug design. | Determining how to use equivariant diffusion models to improve the accuracy of structure-based drug design. |
| SILVR: Guided Diffusion for Molecule Generation by Nicholas et al 2022. | Guided diffusion approach for molecule generation. | To generate molecules with improved control over their characteristics and functionalities, as well exploring guided diffusion methods. |

**CONTEXT DIAGRAM**

The given Figure 4, shows the context diagram of our model. This figure shows how user-inputted data is used to generate new compounds. The user inputs binding site data which is sent to the input system, which then searches for lead compounds that are then sent to the model. The model then generates new compounds which are sent back as output from model to input system and back to the user.


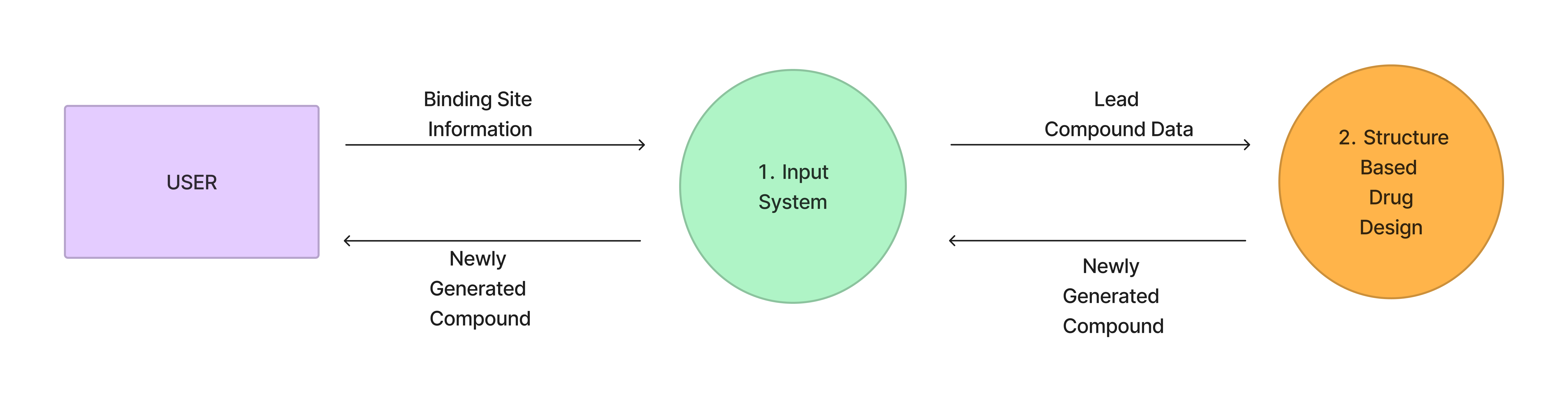


Figure 4: Context Diagram of Model

**DATA FLOW DIAGRAM LEVEL 1**

The given Figure 5, shows the data flow diagram level 1 of our model. This figure shows how user-inputted binding site data flows through the model generating a new compound. The user inputs binding site data, which is then sent to the database through a server. The binding site data is then used to identify lead compounds from the database, which are then sent to the Vectorization module that converts them into a machine-readable format. The data is then sent to a generative model to generate a new compound. This new compound data is then sent back to the server, where the server sends this data back to the user as output.


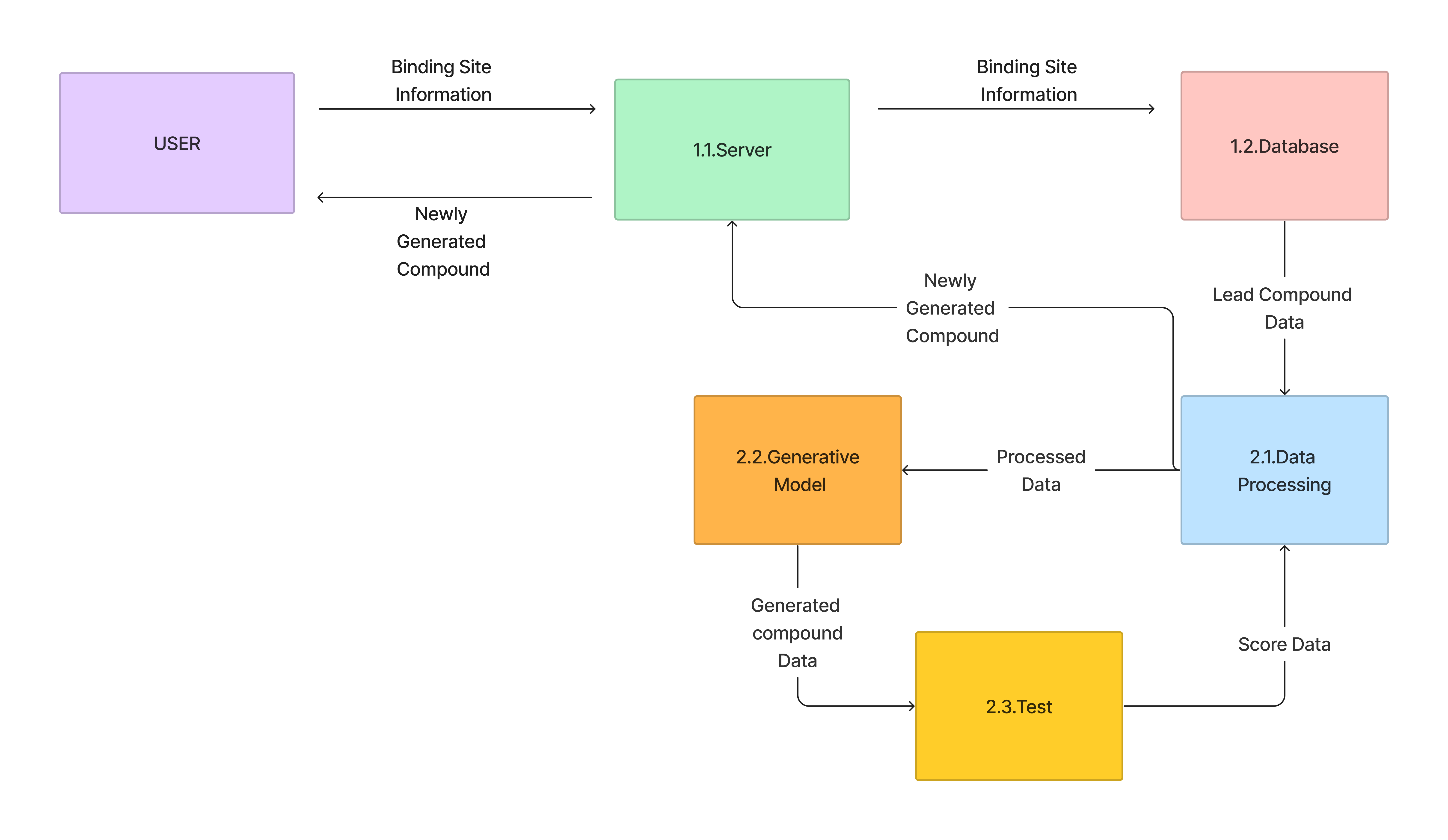


Figure 5: Data Flow Diagram Level 1 of Model

**ENVIRONMENT SETUP**

We used a variety of tools and libraries to build a strong computational environment
to carry out our research and experimentation. Python was the preferred programming
language for our implementation because of its wide range of applications and strong
support in the scientific community.
We used Matplotlib, a robust plotting library with many configurable options, for
both graph production and visualization. This made it easier to create the kind of clear,
perceptive visuals that are necessary for the examination of our findings.
PyTorch, a well-known and dynamic library that provides extensive support for creat-
ing and training neural networks, was our preferred machine learning framework. Taking
advantage of PyTorch’s capabilities, we put our models into practice and adjusted them
to get the best results for our particular needs.
Pandas and NumPy were used in the creation and processing of the dataset. NumPy
enabled array manipulations and numerical operations that were essential for several areas
of our research, while Pandas, a data manipulation toolkit, offered effective tools for
managing structured data.
This well-chosen setting guaranteed a seamless integration of several tools and en-
hanced the repeatability and dependability of our findings. The combination of Python,
Matplotlib, PyTorch, Pandas, and NumPy strengthened our research efforts and enabled
us to establish our project efficiently.

**TESTING AND EVALUATION**

We have performed direct comparison of **CoDNet** with state-of-the-art models such as GSchNet, EDM, EDM+OBabel, MiDi (uniform), and MiDi (adaptive). Its performance is examined through a multi-pronged approach on the QM9 data set.

We have tested for **CoDNet** validity, connected component, novelty, uniqueness, valency, bond lengths, angles.

**Validity:** To judge its ability to produce chemically valid compounds.

**Connected component:** To see how connected the compounds generated by it are to evaluate its ability to capture complex molecular relationships.

**Novelty:** To evaluate its ability to generate new drugs.

**Uniqueness:** To evaluate its ability to generate distinct molecular representation.

**Valency:** The number of chemical bonds an atom can form, visualized as "arms" reaching out to connect with other atoms.

**Bond Length:** The distance between the nuclei of two bonded atoms, typically measured in Angstroms (Å) and pictured as the space between the "arms" connecting the atoms.

**Angles:** The measure of the space between two "arms" connected to a central atom, crucial for determining the 3D shape of a molecule and its interactions with other molecules.

We have evaluated its ability to capture physical properties by calculating the the valency, bond lengths and angles between the atoms of the compounds generated by it.

**FUTURE WORK**

In the future, our primary focus will be on enhancing our model's adaptability by incorporating more extensive conditioning. We aim to customize our model further, ensuring it can better align with diverse requirements. By embracing increased conditioning, we anticipate achieving a higher level of customization, enabling us to address a wider range of applications and refine our model's performance. This strategic evolution has the potential to play a pivotal role in refining drug development, allowing for highly customized medications tailored to individual patient profiles. Our commitment to advancing healthcare technology and patient-centric pharmaceuticals remains unwavering, and we eagerly anticipate the outcomes of our ongoing efforts.

**REFERENCES**

[1] C. Vignac, N. Osman, L. Toni, and P. Frossard, “MiDi: Mixed Graph and 3D Denoising Diffusion for Molecule Generation.” 2023.
